# Management of dyslipidemia in children

**DOI:** 10.1186/1758-5996-1-26

**Published:** 2009-12-08

**Authors:** Sanjay Kalra, Arun Gandhi, Bharti Kalra, Navneet Agrawal

**Affiliations:** 1 Department of Endocrinology, Bharti Hospital, Karnal, India; 2 Department of Endocrinology, Medical College, Mullana, India; 3 Department of Endocrinology, Medical College, Gwalior, India

## Abstract

Dyslipidemia is an important etiologic factor in the development of cardiovascular disease (CVD), which is a leading cause of death worldwide As CVD begins in childhood, and as dyslipidemia is an important risk factor for CVD, screening and treatment of dyslipidemia in adolescents and children becomes an important health matter. This review deals with issues related to screening, diagnosis and treatment of dyslipidemia in children and adolescents.

## Introduction

Dyslipidemia is an important etiologic factor in the development of cardiovascular disease (CVD), which is a leading cause of death worldwide [1]. Studies reveal that CVD begins in childhood [2,3] and endothelial damage has been noted in the first few years of life in children with lipid abnormalities [4,5]. This, associated with unhealthy diet, lack of physical activity and weight gain, [6,7], makes screening and treatment of dyslipidemia in adolescents and children an important target for prevention of CVD and mortality.

In spite of the public health importance of childhood dyslipidemia, comparatively less attention has been paid to the need to screen, identify and treat children and adolescents with dyslipidemia. This review addresses this issue by focusing on the various diagnostic and therapeutic strategies available to correct dyslipidemia in the younger population. It highlights recent guidelines, and concludes with a call for more aggressive management of dyslipidemia in this age group.

## Screening: Clinical Issues

The National Cholesterol Education Program (NCEP) Expert Panel in Blood Cholesterol levels in Children and Adolescents [8] has proposed a two tier approach, vis: a population based and an individual based strategy.

The population based approach relies on health professionals to encourage dietary and lifestyle management, including exercise, healthy weight maintenance, and cessation of smoking. School nurses can be utilized to spread these, and other, messages related to prevention of CVD.

The individual based strategy identifies children at increased risk of premature CVD by clinical and biochemical criteria.

Children ≥ 2 years of age with a first or second -degree relative with documented CVD before age 55 in men and 65 in women should undergo a fasting lipid profile. CVD is defined as any of angina pectoris, peripheral or cerebral vascular disease, myocardial infarction, documented coronary artery disease or sudden death.

Children and adolescents with, evidence of risk factors viz. overweight, hypertension, diabetes, smoking, poor diet, and sedentary lifestyle, should also be screened for dyslipidemia, even if they do not volunteer a positive family history of premature CVD.

## Screening: Biochemical Issues

The serum screening tests should include total cholesterol (TC), high density lipoprotein- cholesterol (HDL), triglyceride (TG), and calculated low density lipoprotein- cholesterol (LDL) levels [8]. LDL is usually calculated using the Friedewald formula (LDL = TC-(HDL+TG/5) [9], but this is not accurate if TG levels are above 400 mg%

A borderline or high result should prompt a repeat test. Classification of reports is based on data obtained from children. 'Borderline' and 'high' correspond to 75^th ^and 95^th ^percentiles, respectively. The acceptable values for TC, LDL, HDL and TG are <170, <110, ≥40 and <150 mg% respectively. Borderline values for TC, LDL and TG are 170-199, 110-129 and 150-499 mg% respectively, with values higher than these qualifying to be labeled 'high' [8].

A random cholesterol sample is enough for children with a parental history of high cholesterol (>200%) without premature CVD. A repeat sample can be tested if the level is >170 mg%. An average value >170 mg% merits a fasting lipid profile, and classification of the individual according to LDL values obtained from the fasting sample.

## Further Evaluation

A child with positive values should be evaluated further [8]. A review of family history should be carried out and a complete history and medical examination done to rule out secondary cause of hypercholesterolemia. Focus should be on drug history, physical activity, dietary patterns and use of tobacco. Physical examination must include a detailed anthropometry, assessment of puberty, goiter and liver size. Signs of insulin resistance such as acanthosis nigricans; xanthomas, xantholasmae, skin tags, palpable arterial walls and gout should be sought.

Laboratory test including liver, renal, thyroid function tests and glucose levels should be ordered and abnormal values managed appropriately.

## Follow Up

Further management plans are based on fasting LDL concentrations [8]. An LDL value <110 mg% is acceptable, and need not be repeated for 5 years in children. Healthy lifestyle practices should be encouraged in all such patients.

An LDL value of 110-129 mg% will classify as 'borderline', and will prompt diet (American Heart Association (AHA) step I) [10] and lifestyle management, as well as a repeat lipoprotein analysis after 1 year. No pharmacological lipid- lowering therapy is indicated in these children or adolescents. The goal is to reach an LDL level with the acceptable range.

In children with an LDL level > 130 mg% one should work up for potential secondary causes, initiate a dietary and lifestyle modification range, and repeat lipid profile after 3 months.

Patient who have reached LDL <130 mg% should continue the same regime, and get a repeat test at 1 year. Those who still have an LDL >130 mg should begin a step II diet, and repeat lipid profile at 3 months.

In case LDL is still above >130 mg% one should continue step II diet [10] and lifestyle management, while recommending additional fiber and plant sterols, with or without lipid lowering drugs. Lipid profile should be done at 3 to 6 monthly intervals.

The initiation of lipid -lowering medication will depend on LDL levels, presence of other CVD risk factors, and family history of premature CVD.

## Dietary Treatment

A diet with <10% calories from saturated fat, 30% calories from fat, and <300 mg/day from cholesterol (AHA step I diet) [8,10] is recommended for all healthy children ≥2 years of age. Intake of polyunsaturated fats, mono-unsaturated fats, omega-3 fatty acids, and high -fiber foods should be encouraged [11,12].

In case this is not successful in achieving cholesterol targets, a more stringent diet plan, the AHA step II diet, is indicated. Dairy saturated fat is reduced to <7% and dietary cholesterol to <200 mg/day, No adverse events have been noted on growth, iron stores, nutrition or well being over a period of 3 to 10 years [13,14] in children following a step II diet.

## Lifestyle Treatment

Children should be encouraged to indulge in 60 minutes or more of vigorous play or aerobic activity per day [15]. Sedentary time should be reduced as possible, with focus on minimizing time spent on television, internet and videogames.

Tobacco and alcohol use should be actively discouraged, and eating disorders identified and corrected as early as possible.

## Drug Treatment

Drug treatment may be necessary to achieve target LDL cholesterol. Pharmacological intervention is recommended in children ≥10 years with poor response to diet and lifestyle therapy, documented for at least 6-12 months. The choice of therapy is influenced by the lipid profile, age, gender and family history of the patient.

Recent guidelines [16,17] have lowered the LDL cutoff levels for treatment, and promoted a more aggressive approach to pharmacological therapy of dyslipidemia in children. Early initiation of drug therapy is warranted in high-risk children and adolescents.

## HMG-Co A Reductase Inhibitors

Statins or HMG-Co A reductase inhibiters are the drug of choice for high LDL in adults, and are increasingly becoming popular in children [16,17]. The safety and efficacy is similar in children and adults [18]. Evidence regarding the safety and efficacy of statins in children and adolescents is now available, over a period of 24 months follow up. Statins lead to LDL-C lowering with minimal adverse effects, without affecting growth, maturation [19], hormonal status and quality of life.

It is recommended that children should be started on low dose of statins, with gradual upward titration if required [3]. Average starting doses will be rosuvastatin 5 mg, atorvastatin 10 mg and simvastatin 20 mg/day, with a close watch on side effects.

Through routine liver function tests and creatine kinase estimations are not recommended in adults, they should be performed in children every 3 to 4 months. Children may experience transient rise in creatine kinase levels after vigorous physical activity. Treating physicians should be aware of possible drug interactions including those with gemfibrozil, cyclosporine, and erythromycin.

Grapefruit juice may increase the bioavailability of simvastatin and atorvastatin by reducing the levels of metabolism in the gut. Children and adolescents prescribed these drugs should be informed about the potential adverse effects of taking grapefruit juice.

Atorvastatin and simvastatin are approved in boys ≥10 years of age and in post menarchal girls, while pravastatin is approved after age 8 years in both genders. Current guidelines encourage the use of statins prior to age 10 in select children with high LDL and strong risk factors [16,17].

## Ezetimibe

Ezetimibe enters the enterohepatic circulation and reduces bile acid reuptake as well as absorption of cholesterol, in a single fixed dose of 10 mg/day. Though its long term use has not been studied in children yet, its favourable safety profile makes it an attractive drug for use in the younger age group, both as monotherapy and in combination.

Ezetimibe is approved in children above 10 years of age, in a dose of 10 mg/day, but studies are very few in number [20,21]. The role of ezetimibe is as an adjuvant to statin therapy.

## Niacin

Niacin is the most potent HDL enhancer available today. Niacin has been used in small series of children [22] and is recommended only as adjunctive therapy in children being supervised by a lipid specialist [8,23].

The availability of a niacin/laropiprant combination which avoids flushing and other side effects associated with conventional niacin therapy may make it more popular in the management of pediatric dyslipidemia.

## Fibrates

Fibrates are used in children with very high triglyceride levels (> 400 mg %) to prevent pancreatitis. Children tolerate this class of drugs well [24,25] but elevation of liver enzymes, gastrointestinal symptoms, and predisposition to cholelithiasis can occur. This class of drugs, including fenofibrate and bezafibrate, has been used in few children [24,25] only.

Combination therapy of fibrates and statins predisposes children (and adults) to an increased risk of rhabdomyolysis, and should be avoided in younger patients.

The NCEP pediatric panel has not recommended any targets for non-LDL cholesterol or for triglycerides [8]. This reduces, in relative terms, the importance of fibrates, which specifically target triglyceride, rather than LDL levels.

## Dietary Supplements

Plant sterols and stanols can be used in children, while monitoring for effects on absorption of fat -soluble vitamins [12].

Omega-3 fatty acids and antioxidant vitamins can be used in hyperlipidemic children but results are controversial [26].

In general, dietary supplements are not encouraged as monotherapy in hyperlipidemic children.

## Bile Acid Sequestrants

Bile acid sequestrants are traditionally thought to be the first line therapy for children with dyslipidemia [26]. This is because they are older agents which have been available for a longer time, had the best evidence base at the time of the NCEP pediatric panel recommendations, and are not systemically absorbed However, cholestyramine is available only as a powder, and colestipol as granules or large tablets. Both are unpalatable, associated with gastro intestinal side effects and fat soluble vitamin and folic acid malabsorption. Compliance to this therapy is poor.

They should be co administered with vitamin D and folic acid.

This class of drugs is now being supplanted by statins as the drug of choice for childhood and adolescent dyslipidemia.

## Teratogenicity

All cholesterol -lowering drugs except omega -3 fatty acids are contraindicated in pregnancy or in women planning pregnancy [8]. Adolescent girls and young women who are sexually active should practice appropriate contraception if they are on statins, fibrates or other cholesterol lowering drugs.

## Limitation of current guidelines

The current guidelines described for screening and managing children and adolescents with dyslipidemia do have some limitations.

Screening is based on family history, which may not be available, or may not be accurate, in all cases. Percentile definitions do not take into account gender, race, ethnicity age and pubertal status. LDL levels alone are used to guide treatment and other risk factors/variables such as HDL cholesterol, non- HDL cholesterol, triglycerides and apoprotein- B are not taken into account.

The changes normally seen in lipid levels during childhood and adolescence are not considered in these guidelines. Cholesterol levels are highest at age 9 to 11 years, decrease throughout adolescence, and increase later on [27,28]. This temporal change may be linked to the transient increase in insulin resistance at the onset of puberty.

## Genetic counseling

Genetic counseling is required in families with familiar hypercholesterolemia. They should be explained the nature of the illness and its inheritance, and the need for appropriate therapy [26]. Effective genetic counseling helps in ensuring improved compliance to therapy, and makes the patient an active partner in monitoring and therapy.

## Conclusion

This review has tried to address the salient issues related to screening and management of dyslipidemia in children. It will sensitize physicians and pediatricians to the need to correct this easily modifiable risk factor, so as to reduce the burden of disease and death in our future generations. Universal, and aggressive, use of the current guidelines is necessary to prevent future CVD.

## Competing interests

The authors declare that they have no competing interests.

## Authors' contributions

SK and AG wrote the initial manuscript. BK and NA conceived the idea. All authors contributed to finding references and correcting the manuscript. All authors read and approved the final manuscript.
